# Array CGH-based detection of CNV regions and their potential association with reproduction and other economic traits in Holsteins

**DOI:** 10.1186/s12864-019-5552-1

**Published:** 2019-03-07

**Authors:** Mei Liu, Lingzhao Fang, Shuli Liu, Michael G. Pan, Eyal Seroussi, John B. Cole, Li Ma, Hong Chen, George E. Liu

**Affiliations:** 1 College of Animal Science and Technology, Northwest A&F University, Shaanxi Key Laboratory of Agricultural Molecular Biology, Yangling, 712100 Shaanxi China; 2 Animal Genomics and Improvement Laboratory, BARC, Agricultural Research Service, USDA, Beltsville, MD 20705 USA; 3 Department of Animal and Avian Sciences, University of Maryland, College Park, MD USA; 4 College of Animal Science and Technology, China Agricultural University, Beijing, 100193 China; 5 Agricultural Research Organization (ARO), Volcani Center, Institute of Animal Science, Department of Quantitative and Molecular Genetics, HaMaccabim Road, P.O.B 15159, 7528809 Rishon LeTsiyon, Israel

**Keywords:** Array CGH, Copy number variation (CNV), Complex traits, Association, Holstein

## Abstract

**Background:**

Copy number variations (CNVs) are structural variants consisting of large-scale insertions and deletions of genomic fragments. Exploring CNVs and estimating their effects on phenotypes are useful for genome selection but remain challenging in the livestock.

**Results:**

We identified 1043 CNV regions (CNVRs) from array comparative genomic hybridization (CGH) data of 47 Holstein bulls. Using a probe-based CNV association approach, we detected 87 CNVRs significantly (Bonferroni-corrected *P* value < 0.05) associated with at least one out of 41 complex traits. Within them, 39 CNVRs were simultaneously associated with at least 2 complex traits. Notably, 24 CNVRs were markedly related to daughter pregnancy rate (DPR). For example, CNVR661 containing *CYP4A11* and CNVR213 containing *CTR9*, respectively, were associated with DPR and other traits related to reproduction, production, and body conformation. CNVR758 was also significantly related to DPR, with a nearby gene *CAPZA3*, encoding one of F-actin-capping proteins which play a role in determining sperm architecture and male fertility. We corroborated these CNVRs by examining their overlapped quantitative trait loci and comparing with previously published CNV results.

**Conclusion:**

To our knowledge, this is one of the first genome-wide association studies based on CNVs called by array CGH in Holstein cattle. Our results contribute substantial information about the potential CNV impacts on reproduction, health, production, and body conformation traits, which lay the foundation for incorporating CNV into the future dairy cattle breeding program.

**Electronic supplementary material:**

The online version of this article (10.1186/s12864-019-5552-1) contains supplementary material, which is available to authorized users.

## Background

Enhancement of dairy sector is required to meet the increasing demands of animal protein in the world. Since Holstein is the largest milk-producing dairy breed, improving its performance, e.g. production, reproduction, growth, and disease resistance, is crucial for the global agriculture. As one of the most important phenotypes, cattle fertility is affected by both genetic and environmental factors [1]. For example, Holstein accounts for 90% of the U.S. dairy population but has experienced severe declines in fertility over the past 50 years. With a national pregnancy rate of only 15%, cows take longer to conceive and also have delayed lactations, both of which lead to a loss of profit for the farmer. Daughter pregnancy rate (Dtr_Preg_Rate or DPR) is a trait that is used to quantify the number of “days open”, or the number of days between the last calving and conception of the cow (− 1% DPR = + 4 days open) [2]. Contemporary Holstein cows take 30 days longer than cows of 50 years ago to successfully conceive. Although DPR has low heritability, the variability of fertility phenotypes among individuals suggests the possibility of improving fertility without severely affecting milk production. Such a possibility is supported by the stabilization of DPR rates since 2005 for both cattle genders [3].

Genomic structural variants are comprised mainly of copy number variation (CNV) in the form of large-scale insertions and deletions, as well as inversions and translocations [4]. CNVs has been generally accepted as a major source for heritable variation [5]. Compared to SNPs, CNVs often involve larger genomic regions and have potentially greater effects on genome function, including changing gene structure and dosage, alternating gene regulation and exposing recessive alleles [6]. In the last decade, CNV has been widely studied in humans, mice and livestock [4, 7–9]. In cattle, several CNV maps have identified large numbers of CNVs using various approaches [10–15]. However, the effective use of CNV as genomic markers for association with diseases and economic phenotypes is impaired by difficulties in accurately detecting CNV and their boundaries [16]. The detection of phenotype-associated CNV is still challenging in livestock. CGH array, SNP array, and DNA sequencing are the three main approaches to detect CNV. Evaluations on their performances have revealed that all the three platforms have their advantages and disadvantages [16, 17]. Compared to SNP chip, CGH array has a series of advantages on CNV detection. For example, CGH array has greater sensitivity to detect small differences in copy number, because it analyzes copy loss and gain variations in a single experiment by measuring the relative hybridization intensity between fluorescently labeled test and a single reference DNA sample, whereas SNP arrays use a population reference. Additionally, CGH array shows better signal-to-noise ratios than SNP arrays, thus many of duplications missed by SNP arrays can be detected by CGH arrays [16]. Moreover, a dense and uniform CGH array can be rapidly synthesized and be customized to target virtually any region of interest (including repeat-rich regions) [18].

Understanding of chromosomal regions or genomic variants associated with complex phenotypes can benefit the genome selection in dairy cattle breeding. Genome-wide association analysis (GWAS) is a powerful method of annotating phenotypic effects on the genome. Much attention has been paid to the identification of quantitative trait locus (QTL) associated with complex traits and underlying molecular mechanisms based on SNPs [19–22]. For example, one of these GWAS that used the Illumina Bovine SNP50K chip to genotype contemporary Holstein cows identified a number of candidate genes for DPR on chr1, chr7, chr18 and chrX; calving ease on chr18; and still birth on chr15 and chr23. Nevertheless, only a few studies have integrated CNVs called from SNP arrays with the economic traits by GWAS. In beef cattle, CNV-based GWAS studies have identified several significant CNVs impacting feed conversion and growth in *Bos taurus* and *Bos indicus* [23–25]. For dairy cattle, several studies have attempted to detect phenotype-associated CNVs using bovine SNP arrays or combination of SNP arrays and sequencing data [26–28]. However, up to now, exploring the relationship between phenotype and CNV detected by array CGH has not been reported in cattle yet.

In this study, we aimed to identify CNVs in U.S. and Israeli Holstein bulls using high-density array CGH data and to explore CNVs associated with 41 production, health, reproduction and body conformation (type) traits. The significant CNVs identified in this work could be utilized as possible molecular markers for genetic improvement program in dairy cattle.

## Results

### CNV identification and distribution

**Fig. 1:**
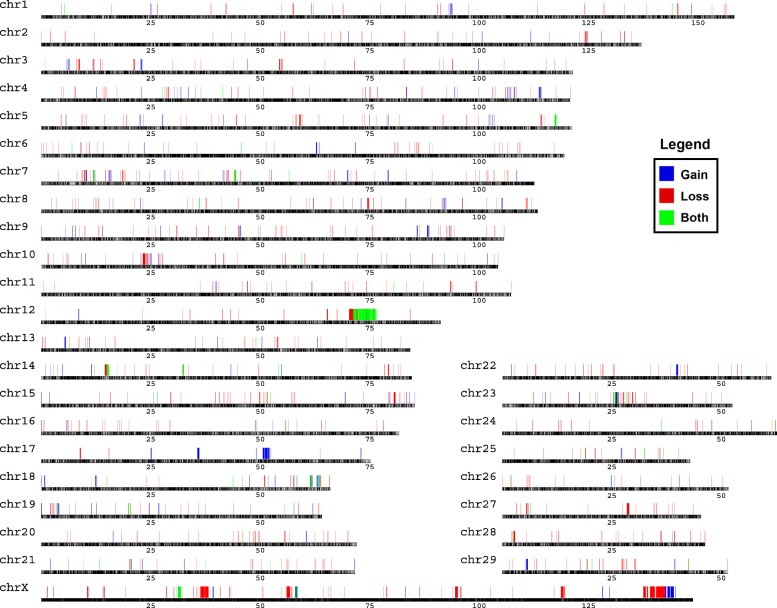
Genomic distribution of CNVRs in 47 Holstein bulls

A total of 1758 CNVs, on the placed chromosomes (chr1–29 and X), were detected in all 47 Holsteins after the quality control (QC) filtering. On average, 37 gain or loss events were present per sample. Across 29 autosomes, we found a varying distribution of CNV (Additional file 1: Figure S1). On chr7, 12, 13, the CNV counts were more than 100 in all samples. The CNV length ranged from 3600 to 2,111,937 bp among 29 autosomes. For average length per CNV, we observed that the ones in chr17 and chr27 were obviously higher than those in other chromosomes. These results were consistent with previous reports in bovine HapMap samples and Nellore cattle, where chr17 also had higher CNV length and chr12 had high CNV count [29]. By merging CNVs, 1043 CNVRs were detected, covering 46,802,944 bp of sequence, i.e., 2.06% of the placed chromosomes (46.8 Mb/2634.4 Mb, Additional file 2: Table S1). Genomic distribution of these CNVRs is shown in Fig. 1, consisting of 702 loss, 270 gain and 71 both (loss and gain within one CNVR) events. As described previously [10], loss events are twice more frequent than gain ones. Also, the CNV length for loss events (26,154,948 bp) is approximately 1.5 and 2 folds longer than that of gain (11,204,359 bp) and both (9,443,636 bp) events, respectively. Although, on the unassigned chrUn, we detected 51 additional CNVRs of 17,976,696 bp, due to the lack of sequence and/or the mapping uncertainty, these CNVRs were not analyzed further.

### Gene annotations for the discovered CNVRs

We next annotated the gene content spanning CNVRs. Based on the gene models for the cattle genome UMD3.1 assembly, we found that 1043 CNVRs within known chromosomes overlapped with 761 Ensembl peptides, corresponding to 322 gene symbols (Additional file 2: Table S1). Using the PANTHER analysis, we observed statistically significant over- or under-representations for multiple Gene Ontology (GO) terms (Additional file 2: Table S2). The enriched GO terms included four molecular function terms (G-protein coupled receptor activity, transmembrane signaling receptor activity, signaling receptor activity, molecular transducer activity), 19 biological process terms (e.g. antigen processing and presentation, chromatin assembly, detection of chemical stimulus involved in sensory perception of smell, sensory perception, detection of stimulus involved in sensory perception), and four cellular component terms (e.g. MHC class II protein complex, integral component of membrane). The observations were consistent with previous CNV analyses in cattle [10, 13]. The gene families important for the bovine MHC (BoLA), ATP-binding cassette (ABC) transporters, defense/innate and adaptive immunity, and signal recognition olfactory receptors have also been observed, supporting the shared GO terms among mammals.

### CNV association analyses

In order to explore the influence of CNV on the complex traits, we conducted association analysis of CNV with complex phenotypes using a mixture model implemented in CNVtools. A total of 297 CNVRs in 29 autosomes have statistically converged for all the 41 studied complex traits. The detailed association results are shown in Additional file 2: Table S3. Among them, 87 CNVRs were significantly associated with at least one trait (*P* < 0.05) after multiple testing correction (the Bonferroni method) (Additional file 2: Table S4). To explain these CNVRs’ impacts on these complex traits, we approximately calculated the proportion of phenotypic variance (i.e., breeding values; PTA) explained by all studied CNVRs and found their average was 0.0376 with a standard deviation of 0.0512 and a median of 0.0172. We clustered these 41 traits based on the *P* values of these 297 CNVRs generated by the association study. In general, the phenotypes were grouped by 3 major types, consistent with the previous result that was based on SNP statistics from single-marker GWAS (https://www.biorxiv.org/content/early/2018/10/02/428227). Body type, production and reproduction traits were grouped separately while the health traits were intertwined with production or reproduction traits (Additional file 1: Figure S2).

### Genes within or near the significant CNVs

We further investigated the genes within or near the significant CNVRs. Among the 87 significant CNVRs, 35 CNVRs overlapped with the coding or flanking (± 5 kb) regions of 47 protein genes. For other CNVRs, the nearest neighboring genes and the distances from the CNVRs were also shown due to the hypothesis of CNV’s long distance effects [6]. To further investigate the potential effects of these CNVs on complex traits, the expression patterns of their overlapped or closest genes were investigated across 91 cattle tissues and cell types based on RNA-seq [Fang et al., 2018, in preparation]. Some genes were widely expressed whereas others were specifically highly expressed (top 3%) in a limited number of tissues (Additional file 2: Table S4). From the results, we noticed that some CNVRs and genes had more striking association(s) with the traits in terms of statistical significance and known relevant biology, making them more likely candidates for causal effects. A brief summary of such CNVRs and genes is listed in Table S4 and described below.

## Discussion

### General discussions about significant CNVRs associated with 4 main trait categories

For the reproduction phenotypes, we detected 35 CNVRs to be associated with eight reproduction traits including Dtr_Preg_Rate, Heifer_Conc_Rate, Cow_Conc_Rate, gestleng, Dtr_Calv_Ease, Sire_Calv_Ease, Dtr_Still_Birth, Prod_Life. Of note, 24 of CNVRs mentioned above were significantly associated with DPR. For example, CNVR423 (chr21: 20,323,800 - 20,338,200) and CNVR120 were associated with both DPR and somatic cell score (SCS), which was similar to the previous SNP-based GWAS study [19]. On the other hand, we found that DPR also shared many common CNVRs (CNVR659, 423, 661,120, 213, 941, 584, and 386) with heifer or cow conception rate. Productive life measures a cow’s longevity in the herd and is affected by production, health and reproduction traits. We identified four CNVRs (CNVR661, 408, 350, 714), which were associated with productive life. Three of them were shared with health traits including livability, displaced abomasum, and metritis, while two (CNVR661 and CNVR714) were shared with fertility traits (DPR and related conception rates) and one (CNVR661) shared with production trait (protein yield). Consistently, previous studies have shown that productive life is more related to health and fertility traits than to production and calving traits [19]. In addition, productive life also shared significant CNVRs with body conformation traits, such as Fore_udder_att, teat length and rump width. For significant CNVRs for production, health, and body conformation phenotypes, please see Additional file 2: Table S4’s notes.

### Three significant CNVR examples

**Fig. 2:**
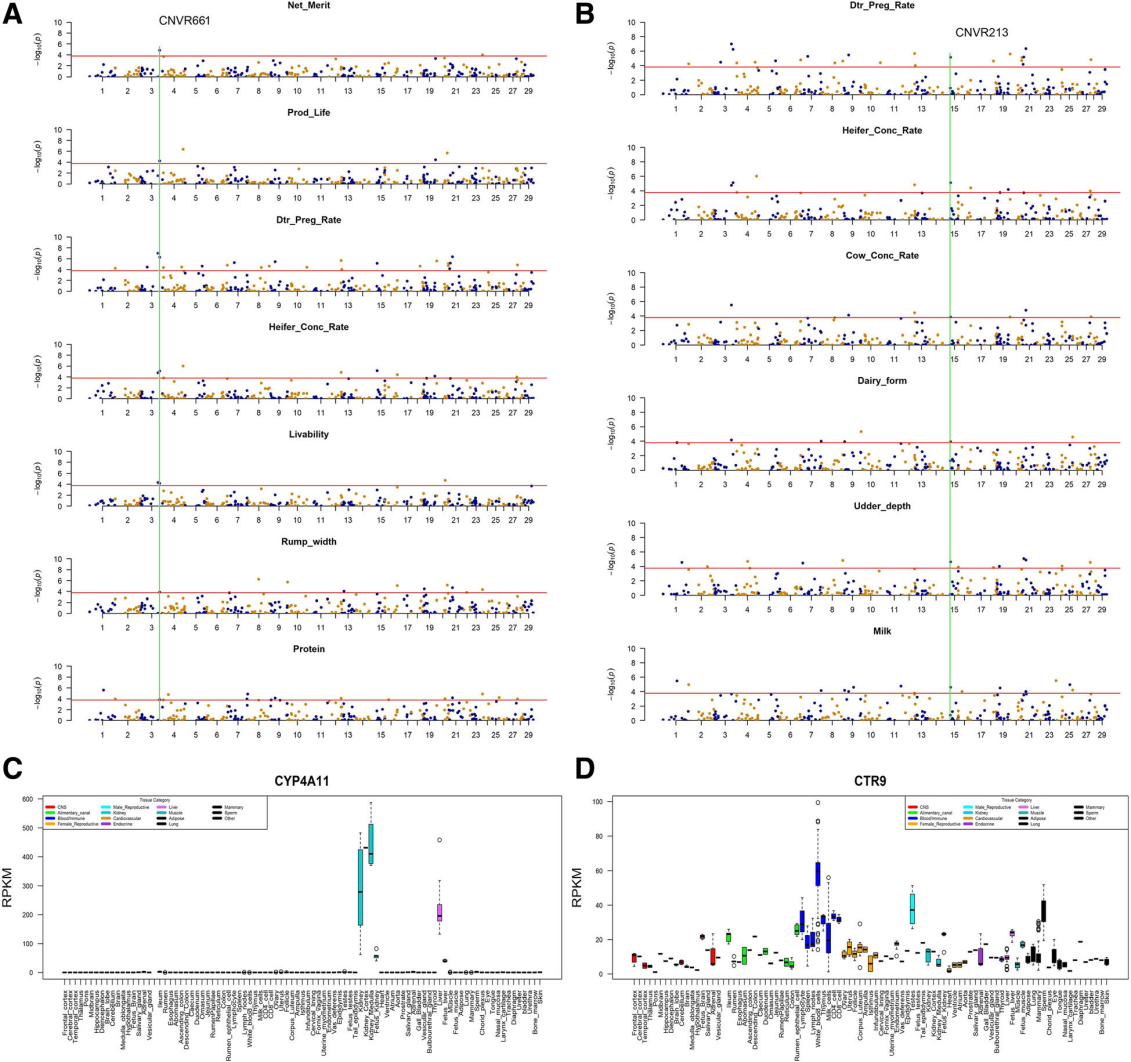
Manhattan plots of example associated CNVs for multiple complex traits and the CNV-overlapped genes **a** CNVR661 (chr3: 99,808,706 - 99,846,316) was significantly associated with seven traits including reproduction (Net_Merit, productive life [Prod_Life], daughter pregnancy rate [Dtr_Preg_Rate], heifer conception rate [Heifer_Conc_Rate], livability), milk production (Protein yield [Protein]), body conformation (Rump_width). **b** CNVR213 (chr15: 42,483,000 - 42,495,000) was markedly associated to six complex traits including reproduction (Dtr_Preg_Rate, Heifer_Conc_Rate, cow conception rate [Cow_Conc_Rate]), body status (dairy form, udder depth), and milk production (Milk yield [Milk]). Negative log10-transformed *P* values from a genome-wide scan are plotted against genomic coordinates on 29 autosomal chromosomes. **c** Expression pattern of *CYP4A11* gene across 91 cattle tissues. **d** Expression pattern of *CTR9* gene across 91 cattle tissues

Among the 87 significant CNVRs, 39 loci were simultaneously related to at least 2 traits, suggesting their pleiotropic effects. Notably, 10 CNVRs were significantly associated with ≥5 traits. For the first example, CNVR661 (chr3: 99,808,706 - 99,846,316) was associated with seven different phenotypes related to production (Protein), female fertility (Dtr_Preg_Rate, Heifer_Conc_Rate), reproductive (Prod_Life, net merit, livability), body conformation (rump width) (Fig. 2A). Compared to a previous study [13], CNVR661 was also harbored by CNVR44 identified in Chinese bulls. In terms of explained proportions of phenotypic variance explained, CNVR661 contributed 5.30% to Dtr_Preg_Rate, suggesting CNVR661 could be an important genetic variance for cattle female fertility. CNVR661 partially overlapped with the coding regions of *CYP4A11* (cytochrome P-450 4A11, chr3: 99,806,653 - 99,820,784), which is a major lauric acid (medium-chain fatty acid) omega hydroxylase in human liver. *CYP4A11* is involved in fatty-acid metabolism, blood pressure regulation, kidney tubule absorption of ions; and can convert arachidonic acid to 20-hydroxyeicosatetraenoic acid (20-HETE). Of interest, *CYP4A11* was specifically highly expressed in kidney and liver according to gene expression atlas (Fang et al., 2018, in preparation) (Fig. 2C). In Chinese cattle, Yang et., al [30] has demonstrated the positive effect of *CYP4A11* copy number on body size traits, which may be due to the dosage effects of *CYP4A11* copies on the gene expression level in liver, kidney, muscle and adipose. These evidences indicated that the strong associations between CNVR661 and multiple phenotypes could involve its effect on the *CYP4A11* function. The second example is CNVR213 (chr15: 42,483,000 - 42,495,000), which may have impacts on reproduction (Dtr_Preg_Rate, Heifer_Conc_Rate, Cow_Conc_Rate), on body status (dairy form, udder depth), and on milk production (Fig. 2B), with effects ranging from 2.27 to 4.96%. Interestingly, *CTR9* (CTR9 homolog, Paf1/RNA polymerase II complex component) was found to locate in CNVR213 and was highly expressed in tissues related to blood immune (e.g. thymus, white blood cells, CD4, CD8 cells), male reproduction (testes), and sperm (Fig. 2D). CTR9, a key component of the PAF1 complex, associates with RNA polymerase II and functions in transcriptional regulation and elongation [31]. PAF1 complex also plays a role in the modification of histones and has multiple functions during transcription by RNA polymerase II [32]. The *CTR9* showed high expression in thymus and immune related cells. CTR9 has been demonstrated to involve in cord blood-associated megakaryopoiesis [33]. These evidences suggested that CNVR213 might have dosage effects on *CTR9* gene expressions in the related tissues, therefore affect CTR9’s regulatory role in reproduction traits. The third example is CNVR758 (chr5: 91,755,000-91,765,800), which explained considerable proportions for Dtr_Preg_Rate (14.30%). We noticed that CNVR758 was located at the upstream of gene *CAPZA3* (the capping actin protein of muscle Z-line alpha subunit 3), which encodes an actin capping protein and is one of the F-actin capping protein alpha subunit family. F-actin-capping proteins play a role in the morphogenesis of spermatid. A previous study has demonstrated that CAPZA3 protein may be important in determining sperm architecture and male fertility [34]. Hence, we speculate that CNVR758 near *CAPZA3* may affect the *CAPZA3* transcription and thus lead to the phenotype effects on bull fertility.

### Previous cattle QTLs overlapped with CNVRs

We also identified CNVRs that spanned potential cattle QTLs and OMIA genes influencing disease susceptibility. Among all CNVRs, 91 CNVRs were overlapped with 553 cattle QTLs (Additional file 2: Table S1). By querying against OMIA, we found 9 CNV-overlapped genes, which were related to coat color, health and diseases in ruminants (such as cattle, yak, goat, sheep), pig, and dog (Additional file 2: Table S1). To further investigate the associations of discovered CNVs with phenotypes, we overlapped CNVRs with previously reported cattle QTLs and observed five CNVRs that were overlapped with QTLs (CNVR659, CNVR120, CNVR26, CNVR459, and CNVR953) (Additional file 2: Table S4). Interestingly, some traits in the QTL database were also present in our CNV-based association results. For example, CNVR659 (chr3: 91,875,073 – 91,890,228) was markedly associated with eight complex traits and overlapped with nine QTLs. Among these traits, Dtr_Preg_Rate, dairy form and udder cleft were observed in both results. Using the genotyped markers, significant QTL for milk production traits have been identified on BTA3 [35]. In Danish Red breed, the QTL for milk yield traits has been found on BTA9 based on SNP markers [36]. Our results showed that CNVR953 (chr9: 56,013,697 - 56,026,693) was associated with milk yield and it overlapped with the milk yield QTL. In Holstein cattle, a preliminary estimate for relationship between CNVs and associated SNPs has showed that approximately three-quarter of CNVs could be captured by LD with nearby SNPs [27]. The consistencies between our results and other SNP-based GWAS results might be in part explained by the linkage between CNVs and tag SNPs that were identified as functional QTL. Therefore, in summary, our study provided multiple possible hypotheses to test for the functional impact of CNV on cattle economically important traits. Many novel CNVs identified in this study could function as potential additional markers.

### Comparison with published results

To date, multiple studies about Holsteins CNV discovery have been published [12, 14, 15, 37]. Nevertheless, CNV discovery studies often produced large calling datasets with certain false positives. We then examined the overlaps of our results with the Holsteins CNVs identified in several previous reports using Illumina BovineSNP50 array [27], BovineHD SNP array [14, 15, 28] and high throughput sequencing technology [26]. Here, only the high confidence CNVRs after filtering by frequency and CNV length in previous studies were used for comparison analysis. In total, 22.13% (216/976) CNVRs within autosomes in our study with a total length of 15,866,448 bp (44.99%) overlapped with the merged CNVRs of the previous five studies by at least 50% overlapping length (Additional file 2: Table S1). Separately, the overlapped CNVR lengths (CNVR count in our study vs. previous study) were 1,900,016 bp (20 vs. 13) compared to 39 CNVRs in [27], 3,739,012 bp (51 vs. 47) compared to 191 CNVRs in [26], 4,165,080 bp (33 vs. 20) compared to 90 CNVRs in [28], 11,419,896 bp (124 vs. 93) compared to 198 CNVRs in [15] and 14,223,966 bp (152 vs. 106) compared to 230 CNVRs with frequency > = 0.05 in [14], respectively (Additional file 2: Table S1). Despite the small sample size of this study, considerable CNVs are still supported by high confidence CNVs in previous Holsteins CNV discovery studies, especially for those with BovineHD SNP array data. Among the 216 common CNVRs, 135 CNVRs were applied for GWAS analysis and 20 of them were observed to associated with Holstein phenotypes (Additional file 2: Table S4). Additionally, to validate the CNV calling results, our previous study has observed a moderate correlation (r = 0.429) between whole genome aCGH probe values and digital aCGH values in six Holstein individuals of this study [11]. Therefore, this study provided further evidences on common CNVs and discovered certain new CNVs.

Previous CNV-based GWAS studies have provided some evidences for CNV impacting phenotypes in Holsteins. Using Illumina BovineSNP50 arrays data, we identified 34 significant CNVs associated with milk production traits with Golden Helix SNP & Variation Suite (SVS) [27]. Based on the BovineHD genotyping data, we found 57 CNVs associated with phenotypes including feed efficiency and feed intake-related traits [28]. Using CNVs identified from both sequencing and the BovineSNP50 array, 15 CNVRs were associated with 7 economically important traits [26]. Compared to them, this study found 87 significant associated CNVs for 28 complex traits. Of note, 20 CNVRs have been supported by common CNVs in previous five studies we investigated and 17 of them were associated with at least two phenotypes. However, only limited parts of our findings have been identified before. For instance, CNVR120, 423, 661 have been reported in previous studies but none reported for their effects on the analyzed traits related to production and body type [26–28]. Therefore, this study described, for the first time, that these CNVRs were associated with DPR and other traits related to production, reproduction, health, and body conformation.

### Advantages and disadvantages of this study

We considered these in four parts. First, this study used a unique array CGH platform. Normally, it is not straightforward to compare CNV results across different platforms. SNP arrays output normalized total intensities (Log R ratio, LRR) and allelic intensity ratios (B allele frequency, BAF), whereas CGH array normally do not consider BAF information. While PennCNV used for SNP arrays can provide accurate calculation of copy numbers when less than 4 copies [26, 38, 39], SNP arrays generally do not have the same sensitivity or resolution of dedicated CGH arrays for high copy number CNV discovery [16, 40]. The SNP chip has the inherent bias coverage against areas of the genome known to frequently harbor CNVs [17, 41], while CGH arrays shown better sensitivity signal-to-noise ratios and specificity, probably as a consequence of longer probes on the array CGH platform. Additionally, many CNVs missed by SNP arrays but detected by CGH arrays are in segmental duplication (SD) regions, which could be due to a combination of differences in probe coverage and the type of reference samples used. Using the single reference, the CGH arrays have greater sensitivity to detect small differences in copy number (e.g., 4 vs. 5) [16]. However, different reference samples may pose a problem in the detection of CNVs and result in the different relative copy numbers among test individuals. Thus, it is important to understand these tradeoffs and use the same reference sample within one study [41].

Secondly, we used different CNV calling algorithms. Multivariate method of SVS was used for SNP arrays in [27, 28], while the segment-calling algorithm (SegMNT) was used for array CGH in this study. Although both use similar segmentation algorithms, the multivariate method is designed for detecting small common CNVs based on multi-sample. As described in the SVS manual, the multivariate method (the pooled marker-level testing across samples) carries out association testing first between the phenotypes and raw intensities at the level of the individual marker, and then aggregates neighboring test results to identify CNVs associated with the phenotype [41]. For CGH-segMNT analysis, the Roche NimbleGen segMNT algorithm was used to call CNV segments in each animal compared to the reference animal. The segMNT algorithm identified copy number changes using a dynamic programming process that minimizes the squared error relative to the segment means, which showed increased accuracy and performance [42]. As for CNVtools method, robust quantitative trait association tests of CNVs were performed based on LRR of probes within each CNV regions. CNVtools then combined the information across a small number of CNV probes to obtain a one-dimensional signal using principal component and Bayesian information criterion for each sample. A copy number genotype was assigned to each locus for each individual to test for genetic association with a quantitative trait based on a standard regression approach [43].

Thirdly, we examined a larger number of complex traits for association analysis. Previous studies only investigated 5 milk production traits [27], 10 production or reproduction traits [28], and 7 production, functional and type traits in [26]. With more phenotypes than others, our study performed the CNV-based GWAS study for many complex traits for the first time and provides some new potential markers and promising information for dairy cattle breeding.

Finally, although this study unravels some reasonable and intriguing results, we must acknowledge that given that the limited sample size (*n* = 39) due to the high cost for array CGH, some associations for certain traits in this study could be less reliable. Therefore, further validation by other methods like long reads sequencing technology and larger sample size is necessary in the future.

## Conclusions

To our knowledge, this study is one of the first GWAS for multiplex traits using array CGH based CNV detections in Holstein. Our results identified dozens of CNVs and provided the candidate genes contributing to production, fertility, health and type traits in Holsteins. Characterization of CNV-related economic traits is important for marker-assisted selection and can lay the foundation for further study of the CNV functional impacts on genomic features and on animal performances. The new associated CNVs identified in this research can supply the additional resource for dairy cattle breeding program beyond the previous GWAS studies purely based on SNP markers.

## Methods

### Sample selections

We sampled 47 Holstein bulls based on their divergent Daughter Pregnancy Rates. Among them, seven bulls were from Israel and 40 bulls were contemporary U.S. Holsteins. The source of the extracted DNA was semen from the Cooperative Dairy DNA Repository (CDDR at Beltsville, MD, USA). SNP genotypes of those animals have been included in the routine genomic evaluation program in the United States.

### Identification of cattle CNVs using array CGH

We performed array CGH using the sequenced Hereford cow L1 Dominette’s blood DNA (reference sample) and 47 Holstein bulls’ semen DNA (test sample) on the whole-genome high-density CGH arrays (NimbleGen custom-made cattle CGH 2.1 M arrays, Roche NimbleGen, Madison, WI). The CGH 2.1 M array containing 2,166,464 oligonucleotide probes (with an average interval of 1.2 kbp between probes, NCBI GEO accession no. GPL11314) were designed based on UMD3.0 and fabricated as previously described [18].

Standard genomic DNA labeling (Cy3 for samples and Cy5 for references), hybridizations, array scanning, spatial correction, and data normalization were performed as previously described [10]. The self-to-self control hybridization was performed using the reference sample (Dominette). The genomic variations were represented by gains and losses of normalized fluorescence intensities relative to the reference. The initial data analysis (normalization and segmentation) was performed using the segMNT algorithm of NimbleScan v2.6 software [42]. We selected a set of conservative calling criteria for the final set of high-confidence CNVs, requiring alternations of 0.5 log2 ratios over five neighboring probes (0.5_5), under which no false-positive was found for self–self-control hybridizations. Since all test samples were from bulls (one X chromosome) and our reference was a cow (two X chromosomes), we shifted the chrX baselines to negative values [10]. We conservatively defined the CNV call filtering criteria to reduce false-positives called in the reference DNA self-to-self hybridizations and filtered out likely false CNV using the strict threshold criteria of length < = 1 Kb and > = 5 Mb. After filtering, CNV regions (CNVRs) were determined by aggregating overlapping CNVs identified across all samples [14].

### Gene annotation analysis and overlapping with QTL

Genic content of cattle CNVRs was screened using RefGene annotation file in UCSC database (http://hgdownload.soe.ucsc.edu/goldenPath/bosTau6/database/). To detect potential genes within CNVR, we defined the ‘overlap’ as more than 1 bp in common between the CNV region and the genomic region (including the 3-Kb flanking regions both up- and downstream) of a given gene. QTL database was downloaded from animal QTL database (http://www.animalgenome.org/cgi-bin/QTLdb/index). Considering overly large confidence intervals for some QTL, we filtered out the QTL with confidence intervals > 30 Mb and used a strict threshold to define the overlap as at least 50% of the CNV length were covered by QTLs [27], as detected using Bedtools.

Gene ontology (GO) enrichment analysis was performed using PANTHER with the bovine gene list. We only considered terms with gene count more than 5 and *P*-value < 0.05, after the Bonferroni correction for multiple testing. To explore gene-containing CNV’s potential functional impacts, we queried Online Mendelian Inheritance in Animals (OMIA) database to find genes which could be associated with the inherited disorders and/or other traits. Moreover, we investigated the Ensembl genes within or closest to the significant associated CNVRs and explored their expression patterns in cattle tissues (Fang et al. in preparation).

### Phenotypes

By querying the CDCB database, we retrieved phenotypes with high reliability for 39 contemporary U.S. Holstein bulls and used them for the association analyses in this study. Traditional predicted transmitting abilities (PTAs) were calculated for 41 complex phenotypes, including 18 body conformation traits, 8 health traits, 9 reproduction traits, and 6 production traits. The production traits include milk yield (Milk), fat yield (Fat), protein yield (Protein), fat percentage (Fat_Percent), protein percentage (Pro_Percent), and net merit (Net_Merit). The reproduction traits include cow’s longevity (productive life, [Prod_Life]), calving (service-sire calving ease [Sire_Calv_Ease], daughter calving ease [Dtr_Calv_Ease], service-sire still birth [Sire_Still_Birth], daughter still birth [Dtr_Still_Birth]), fertility (daughter pregnancy rate [Dtr_Preg_Rate], heifer conception rate [Heifer_Conc_Rate], cow conception rate [Cow_Conc_Rate], and gestation length [gestleng]). The health traits include somatic cell score (SCS), Hypocalcemia (CALC), Displaced abomasum (DSAB), Ketosis (KETO), Mastitis (MAST), Metritis (METR), Retained Placenta (RETP), and livability. The body conformation (type) traits include final score, stature, strength, dairy form, foot angle, rear legs (side view) [Rear_legs(side)], body depth, rump angle, rump width, fore udder attachment (Fore_udder_att), rear udder height (rear_ud_height), udder depth, udder cleft, front teat placement (Front_teat_pla), teat length, Rear legs(rear view) [Rear_legs(rear)], feet/legs score (Feet_and_legs), rear teat placement (Rear_teat_pla). These PTA were predicted additive genetic effects after removing fixed non-genetic effects, and the reliabilities of the PTA were used to quantify the amount of information available for different individuals.

### CNV association analyses

Each CNV was analyzed for the association with each complex trait using the R package CNVtools separately, which implements a mixture model [43]. Briefly, within each CNVR, the normalized signals (LRR) of multiple probes were combined to obtain a one-dimensional signal for each sample using a principle component method, implemented in the CNVtools function *apply.pca*. Through clustering the PCA transformed data (first 3 components), a copy number genotype (i.e., 1, 2, 3) was then assigned to animal for the association testing based on a standard regression approach [43]. The CNVRs that were successfully statistically converged were further considered. The multiple testing was corrected using the Bonferroni method. Explained proportions of phenotypic variances for all studied CNVRs were approximately calculated as the squared correlation between the one-dimensional PCA signals obtained from *apply.pca* function and phenotypes (i.e., PTA).

## Additional files

Additional file 1: **Figure S1.** Characteristics of CNV distribution on each autosome. A. Distributions of CNV length per individual. B. Distributions of CNV count. **Figure S2.** Hierarchical clustering of 41 complex traits based on *P* values from association results between CNVRs and phenotypes. Pearson correlation was used to measure distances. Different colors represent various types of phenotype traits. (PDF 294 kb)

Additional file 2: **Table S1.** CNVRs identified in this study and overlapping with QTL, OMIA, and CNVRs in previous studies. **Table S2.** Gene Ontology terms generated by PANTHER analysis. **Table S3.** Results of GWAS between CNVRs and 41 phenotypes of interest in 39 Holstein cattle. **Table S4.** Characterizes for significant CNVs associated to Holstein phenotypes. (XLSX 395 kb)

## Abbreviations

20-HETE: 20-hydroxyeicosatetraenoic acid
aCGH: array comparative genomic hybridization
BAF: B allele frequency
CALC: Hypocalcemia
CAPZA3: capping actin protein of muscle Z-line alpha subunit 3
CNV: Copy number variation
CNVR: Copy number variation region
Cow_Conc_Rate: cow conception rate
CTR9: CTR9 homolog, Paf1/RNA polymerase II complex component
CYP4A11: cytochrome P-450 4A11
DPR: daughter pregnancy rate
DSAB: Displaced abomasum
Dtr_Calv_Ease: daughter calving ease
Dtr_Preg_Rate: daughter pregnancy rate
Dtr_Still_Birth: daughter still birth
Fat: fat yield
Fat_Percent: fat percentage
Feet_and_legs: feet/legs score
Fore_udder_att: fore udder attachment
Front_teat_pla: front teat placement
gestleng: gestation length
GO: Gene Ontology
GWAS: Genome-wide association analysis
Heifer_Conc_Rate: heifer conception rate
KETO: Ketosis
LRR: Log R ratio
MAST: Mastitis
METR: Metritis
Milk: milk yield
Net_Merit: net merit
Pro_Percent: protein percentag
Prod_Life: productive life
Protein: protein yield
PTA: predicted transmitting ability
QC: quality control
QTL: quantitative trait loci
Rear_legs(rear): Rear legs (rear view)
Rear_legs(side): rear legs (side view)
Rear_teat_pla: rear teat placement
rear_ud_height: rear udder height
RETP: Retained Placenta
SCR: sire-conception-rate
SCS: somatic cell score
SD: segmental duplications
Sire_Calv_Ease: service-sire calving ease
Sire_Still_Birth: service-sire still birth
