# Supplementary figures and images for: Array CGH-based detection of CNV regions and their potential association with reproduction and other economic traits in Holsteins
Source: BMC Genomics. 2019 Mar 7;20:181. doi: 10.1186/s12864-019-5552-1 (PMC6407259; doi:10.1186/s12864-019-5552-1)

**A**

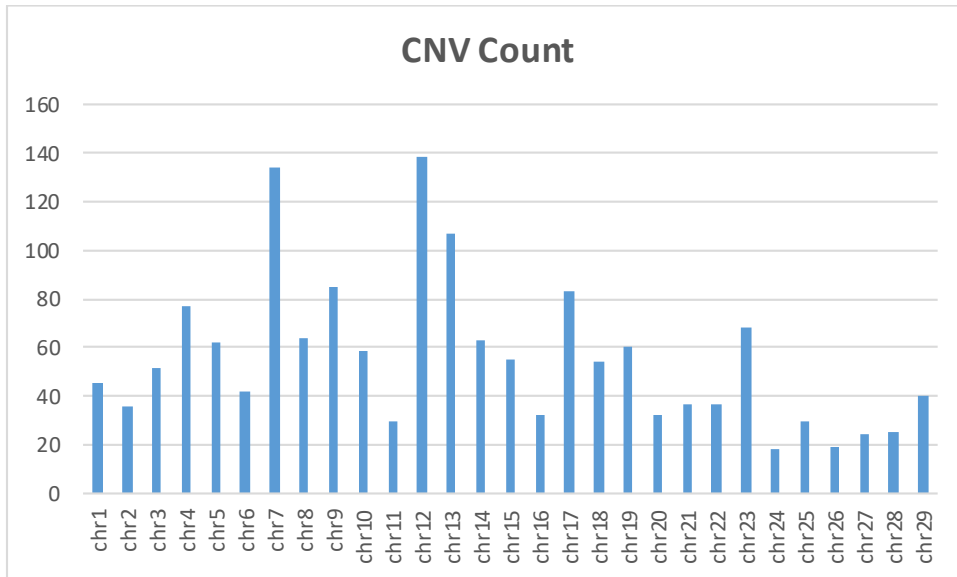

**B**

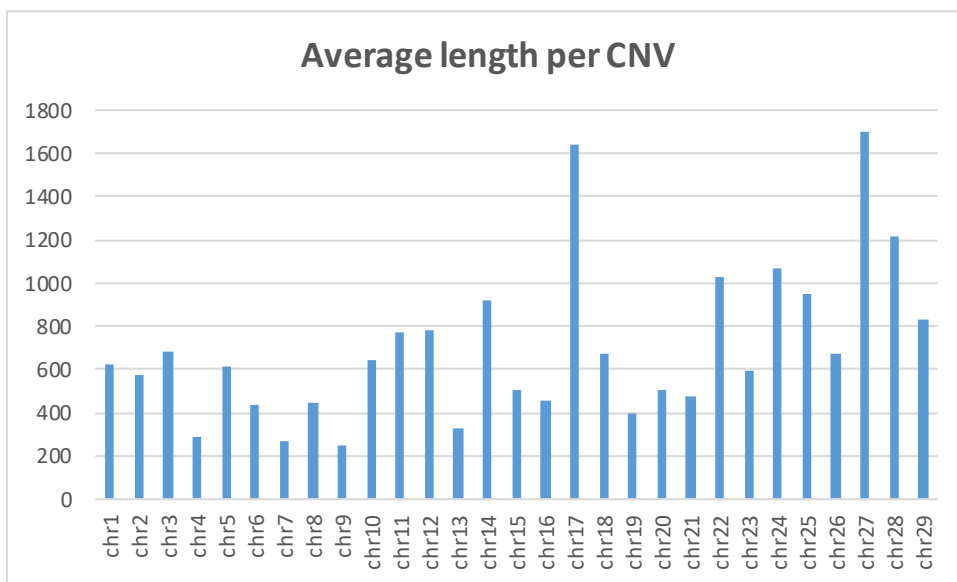

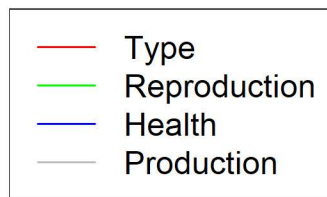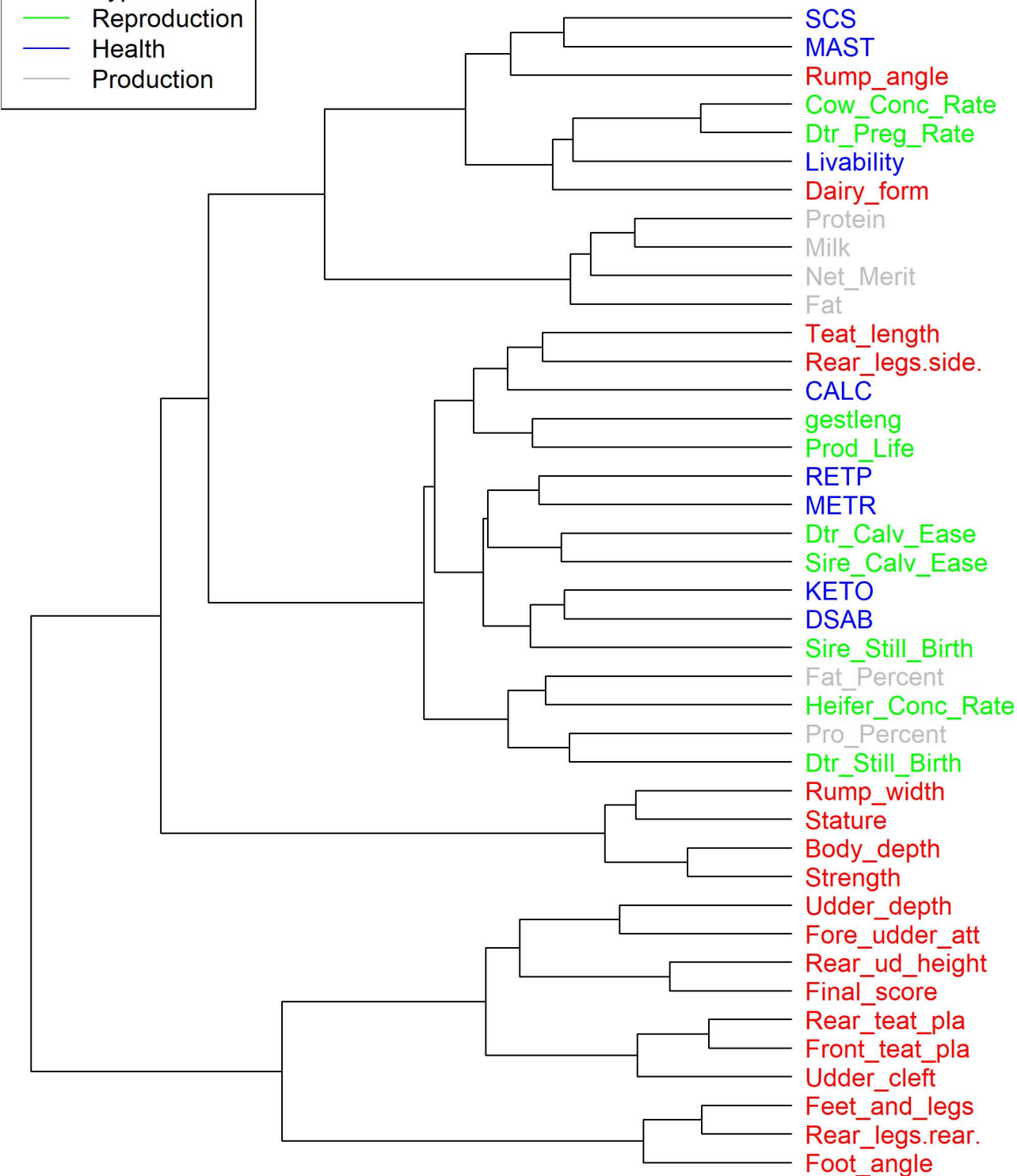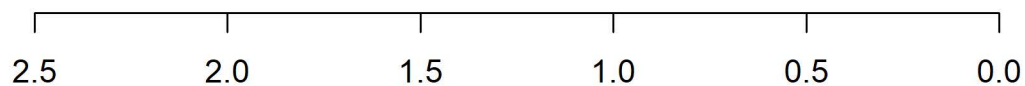

Supplement: Supplementary file 1 — Figure S1. Characteristics of CNV distribution on each autosome. A. Distributions of CNV length per individual. B. Distributions of CNV count. Figure S2. Hierarchical clustering of 41 complex traits based on P values from association results between CNVRs and phenotypes. Pearson correlation was used to measure distances. Different colors represent various types of phenotype traits. (PDF 294 kb) [file 12864_2019_5552_MOESM1_ESM.pdf]
